# Candidate Gene-Based Association Study of Antipsychotic-Induced Movement Disorders in Long-Stay Psychiatric Patients: A Prospective Study

**DOI:** 10.1371/journal.pone.0036561

**Published:** 2012-05-15

**Authors:** P. Roberto Bakker, Egbert Bakker, Najaf Amin, Cornelia M. van Duijn, Jim van Os, Peter N. van Harten

**Affiliations:** 1 Psychiatric Centre GGZ Centraal, Amersfoort, The Netherlands; 2 Center for Human and Clinical Genetics, Leiden University Medical Center (LUMC), Leiden, The Netherlands; 3 Department of Epidemiology, Erasmus MC, Rotterdam, The Netherlands; 4 Department of Psychiatry and Psychology, South Limburg Mental Health Research and Teaching Network, EURON, Maastricht University Medical Centre, Maastricht, The Netherlands; 5 Department of Psychosis Studies, Institute of Psychiatry, King’s Health Partners, King’s College London, London, United Kingdom; Baylor College of Medicine, United States of America

## Abstract

**Objective:**

Four types of antipsychotic-induced movement disorders: tardive dyskinesia (TD), parkinsonism, akathisia and tardive dystonia, subtypes of TD (orofacial and limb truncal dyskinesia), subtypes of parkinsonism (rest tremor, rigidity, and bradykinesia), as well as a principal-factor of the movement disorders and their subtypes, were examined for association with variation in 10 candidate genes (*PPP1R1B*, *BDNF*, *DRD3*, *DRD2*, *HTR2A*, *HTR2C*, *COMT*, *MnSOD*, *CYP1A2*, and *RGS2*).

**Methods:**

Naturalistic study of 168 white long-stay patients with chronic mental illness requiring long-term antipsychotic treatment, examined by the same rater at least two times over a 4-year period, with a mean follow-up time of 1.1 years, with validated scales for TD, parkinsonism, akathisia, and tardive dystonia. The authors genotyped 31 SNPs, associated with movement disorders or schizophrenia in previous studies. Genotype and allele frequency comparisons were performed with multiple regression methods for continuous movement disorders.

**Results:**

Various SNPs reached nominal significance: TD and orofacial dyskinesia with rs6265 and rs988748, limb truncal dyskinesia with rs6314, rest tremor with rs6275, rigidity with rs6265 and rs4680, bradykinesia with rs4795390, akathisia with rs4680, tardive dystonia with rs1799732, rs4880 and rs1152746. After controlling for multiple testing, no significant results remained.

**Conclusions:**

The findings suggest that selected SNPs are not associated with a susceptibility to movement disorders. However, as the sample size was small and previous studies show inconsistent results, definite conclusions cannot be made. Replication is needed in larger study samples, preferably in longitudinal studies which take the fluctuating course of movement disorders and gene-environment interactions into account.

## Introduction

Antipsychotics are the central pillar in the treatment of psychotic disorder. However, these agents can induce movement disorders, which are associated with social stigmatization, physical disabilities and poorer quality of life. They also contribute to non-compliance, which results in an increased risk of psychotic relapse [1]–[3]. Therefore, identification of patients that are prone to these side effects would be of clinical value. Antipsychotic-induced movement disorders [4], [5] can be classified, on the one hand, into acute syndromes, that appear within hours/days or weeks after initiating antipsychotic treatment or increasing the antipsychotic dose (or cessation of anticholinergics), e.g. parkinsonism and akathisia, and, on the other hand, tardive syndromes, that develop after months or years of treatment with antipsychotics such as tardive dyskinesia (TD) and tardive dystonia. Initially, the term ‘tardive’ (delayed) was introduced to emphasize the late-onset types of movement disorders occurring during antipsychotic use. Yet the definition of tardive disorders in the current study emphasizes their persistence, which is clinically more important than their late-onset [5], [6]. Given that combinations of acute and chronic movement disorders occur in patients undergoing long-term treatment with antipsychotics, prediction models should include both syndromes, i.e., the four major types of movement disorders (TD, parkinsonism, akathisia and tardive dystonia).

Family studies suggest an important genetic component to the risk for movement disorders [7]–[12]. A recent meta-analysis on the prevalence of dyskinesia and parkinsonism reported spontaneous dyskinesia and parkinsonism in antipsychotic naïve patients with schizophrenia, and a higher prevalence of dyskinesia and parkinsonism in healthy family members of patients with schizophrenia, compared to matched controls [13].

Pharmacogenetic studies may identify genetic risk factors which underlie individual differences in response to antipsychotics [11], [14], [15], in theory paving the way for individually tailored medication prescriptions [16]. Knowledge of a minimal number of genetic susceptibility loci in candidate genes and demographic, clinical and drug-related risk factors would help the clinician to make a rational treatment choice.

It can be hypothesized that specific subtypes of movement disorders are more suitable for genetic analysis than a general movement disorder syndrome, as subtypes may better reflect the underlying biological heterogeneity in complex syndromes.

The phenotypes under study were TD, parkinsonism, akathisia, and tardive dystonia, subtypes of TD (orofacial and limb truncal dyskinesia), subtypes of parkinsonism (rest tremor, rigidity, and bradykinesia), as well as a principal-factor of the movement disorders and their subtypes.

The 10 candidate genes were *PPP1R1B*, *BDNF*, *DRD3*, *DRD2*, *HTR2A*, *HTR2C*, *COMT*, *MnSOD*, *CYP1A2*, and *RGS2* (Text S1). The choice of these genes was hypothesis-driven, under the common disease/common variant (CDCV) hypothesis, which proposes that common diseases may be caused by common genetic variants [17]–[20].

The aim of the current study was to determine the association between movement disorders and variations in these 10 candidate genes.

The prospective design of the current study extends hitherto cross-sectional work in the pharmacogenetic field of antipsychotic-induced movement disorders. Indeed, prospective assessment of fluctuating (repeated) movement disorders measures the phenotype more specifically and that increases the validity of the associations between movement disorders and risk factors.

## Methods

### Ethics Statement

The protocol was approved by the standing Institutional Review Board, ‘Medisch-ethische Toetsingscommissie Instellingen Geestelijke Gezondheidszorg’ (Review Board for Human Research in Psychiatry), the Netherlands [protocol number 377].

Written informed consent was obtained from each patient, hence, consent obtained from the next of kin was not necessary and not recommended by the Review Board for Human Research in Psychiatry.

### Subjects

A 4-year prospective naturalistic study (July 2003–May 2007) was conducted with 209 patients with chronic mental illness in order to determine the genetic risk factors of the four major types of movement disorders (TD, parkinsonism, akathisia, and tardive dystonia), subtypes of TD and parkinsonism, as well as a principal-factor of the movement disorders and their subtypes. To this end, a cohort was drawn from a general psychiatric hospital (GGZ Centraal, Amersfoort, the Netherlands). Full details of the study design and movement disorders have been published previously [21] (Bakker and colleagues, submitted). The cohort was representative of the population of patients with the most severe chronic mental illness requiring long-stay care, given that the hospital serves an epidemiological catchment area, is the only institute providing this type of care in this area, and patients were selected from a comprehensive list of all inpatients.

Of the patients assessed at baseline (N = 207) 93.7% (n = 194) had at least one follow-up and 59.4% (n = 123) had two follow-up assessments. Loss to follow-up was due to patients who were difficult to trace after leaving hospital, died or refused assessment after inclusion.

### Assessment

Patients were examined by a trained psychiatrist (PRB), using a standard protocol, described by van Harten and colleagues [22]. In addition, subtypes of movement disorders were assessed using (i) the Abnormal Involuntary Movement Scale (AIMS) [23], [24] with items 1–4 for orofacial and items 5–7 for limb truncal dyskinesia, (ii) the Unified Parkinson Disease Rating Scale (UPDRS) [25] with item c3–c4 for ‘rest tremor’ (rest tremor, and action/postural tremor of hands); item c5 for rigidity; and items c1, c2, c6–c12, and c14 for bradykinesia. This approach has been described previously by 3 members of our research team (AAH, JvO and PvH) [26]–[28].

As movement disorders likely share genetic liability, a genetic association between the combined movement disorders and candidate genes is also required. To determine the association between the combined movement disorder and variation in 10 candidate genes, a principal-factor of the four major types of movement disorders and subtypes of TD and parkinsonism was calculated with the FACTOR procedure in the STATA statistical program [29].

Based on the literature published between 1976 and August 2011, we selected 10 candidate genes (Table S1 and Text S1) that (i) are involved in the dopaminergic and serotonergic systems which have been implicated in the development of movement disorders, and the gene coding for the free radical scavenging enzymes like manganese super oxide dismutase (*MnSOD*) based on the hypothesis of neuronal degeneration owing to toxic effects of free radicals on TD. Genes involved in the glutamatergic system that may also contribute to cumulative neural damage, were not selected as the extensive number of receptors in this system, like metabotropic receptors (mGluRs) and ionotropic receptors (iGluRs), merit separate analysis.

In addition, variables possibly affecting risk were extracted from patients’ case notes including age, sex, BMI, self-reported handedness, diagnosis according to DSM-IV, ethnic group (classified as white and non-white), duration of hospitalization and history of electroconvulsive therapy (ECT). Negative symptoms were rated using the negative symptom subscale of the Positive and Negative Symptom Severity (PANSS) scale [30]. The MINI sections for alcohol and drug use were administered, and information on tobacco intake (yes/no, number of cigarettes, cigars, etc; descriptors such as ‘light’, ‘mild’, ‘heavy’ and ‘normal’ use of tobacco) was collected. At baseline and at each follow-up assessment, current use of antipsychotic and anticholinergic medication was collected, and the global symptom rating of the Clinical Global Impression – Schizophrenia severity of illness (CGI-SCH SI) scale was completed. All clinical assessments were carried out by a psychiatrist (PRB). Information on current use of the above medication was collected from the hospital and outpatient pharmacy databases.

The diagnosis ‘schizophrenia’ hereafter refers to DSM-IV codes 295.30, 295.10, 295.20, 295.90, 295.60, 295.70, and other diagnoses of ‘psychotic disorder’ to 295.40, 297.1, 298.8, 298.9.

### DNA Extraction, Genotyping

Two 10 ml EDTA tubes of peripheral blood were drawn from participants, and genomic DNA was extracted from leucocytes by Autopure LS method (Qiangen) according to the manufacturer’s protocols. We genotyped 31 SNPs (TaqMan® SNP Genotyping Assays method, Applied Biosystems, Foster City, California, USA) in 10 candidate gene regions, including SNPs previously reported as associated with movement disorders and schizophrenia.

### Statistical Analyses

#### Hardy weinberg equilibrium

Only SNPs were included in the analyses that were not significantly outside Hardy-Weinberg Equilibrium (HWE) (p>0.05) in (i) the complete control sample (for a dichotomous trait) or (ii) the complete study sample (for a continuous trait). For the three SNPs in the X-chromosomal *HTR2C* gene, departure from HWE was not calculated.

Departure from the HWE was calculated with the GENASS and GENHW procedures in the STATA statistical program [29] for (i) the dichotomously defined persistent forms of movement disorders separately in both patients (with one movement disorder) and controls (without that movement disorder), respectively. Case definition of a persistent movement disorder was based on 2 consecutive assessments over a period of minimally 3 months, and required that individuals met case definition criteria at two consecutive assessments (hereafter: persistent movement disorder), meeting the requirements of Schooler and Kane’s criteria for persistent movement disorder [31], and (ii) the combined group of patients and controls, as continuous measures cannot be separated in both patients and controls.

#### Association tests for single SNPs

Only continuous movement disorder outcomes were used, given that continuous measures better handle the variability of movement disorders and generate more statistical power than cut off points [32], [33]. Genotype and allele frequency comparisons were performed with multiple regression methods for continuous movement disorders, using the Armitage trend test, with the major allele (from our dataset of 168 selected white patients) as reference. The Armitage trend test assumes an additive effect by both alleles on the trait of interest, i.e. the mean effect on the trait by the heterozygous genotype (Major-Minor) is halfway the effects of the two homozygotes. (Major-Major and Minor-Minor).

#### Regression analyses

The regression analyses were conducted with movement disorder measures at a single assessment (hereafter: fluctuating movement disorder). The reason for this was that movement disorders constantly fluctuate over time, so that inclusion in the regression of their repeated single-occasion measures allowed for calculation of associations between one movement disorder with the other over time. As the study design comprised repeated measures nested in the same patient, clustering of observations in individuals needed to be corrected for. Therefore, multilevel random regression was used with the measurement occasion (baseline and two follow-ups) at level 1, and subjects at level 2, with the XTREG MLE routine of the STATA statistical program [29]. Associations with explanatory variables were expressed as beta coefficients representing the change of continuous movement disorder outcome with 1 unit change of the exposure variable.

Using the dataset of 168 selected white patients, associations with predictors were adjusted for *a priori*, movement-disorder specific covariates as follows (Bakker and colleagues, submitted) age was adjusted for in the model of TD and TD subtypes; age and total antipsychotic use was adjusted for in the model of parkinsonism and its subtypes, and no covariates were introduced in the models of akathisia, tardive dystonia and the principal-factor.

Power calculations were performed using the Quanto program version 1.2.4 (http://hydra.usc.edu/gxe).

#### Correction for multiple testing

In order to correct for multiple testing of single SNP tests, the Simes modification of the Bonferroni multiple-testing procedure was performed to control the False Discovery Rate (FDR) [34]. Bonferroni correction is too conservative if tests are not independent of each other (as in this case when there is LD between markers); in this case FDR represents a less conservative alternative. We used the MULTPROC procedure in the STATA statistical program [29] for FDR calculation, and then the SMILEPLOT procedure calling MULTPROC to build a smile plot. A smile plot summarizes a set of multiple analyses, similarly as a Cochrane forest plot summarizes a meta-analysis, and separates by reference line rejected and non-rejected p-values (on a reverse log scale against the corresponding parameter estimates).

#### Defined daily dose

Antipsychotic doses were converted to defined daily dose (DDD), for which we refer to our previous publications [21] (Bakker and colleagues, submitted). Anticholinergic medication was modeled as a dichotomous variable (yes/no).

## Results

### Sample Characteristics

Over the period of observation (mean = 1.1 years, SD = 0.64), of the 209 patients included at baseline, 207 participated in the study. One patient developed a brain tumor, another patient died after inclusion. All patients had a history of cumulative antipsychotic intake of minimally 1 year. Attrition rate was low at 9.8% over a 4-year period.

Of the 207 patients, with chronic psychiatric illness requiring long-term admission, 199 participated in the genetic study. To prevent ethnic stratification resulting in spurious associations owing to differences in allele frequencies and risk of movement disorders, only white patients, representing the most prevalent group (168 = 84.4%), were included in the analysis. At baseline, mean age expressed in years was 48.8 (SD 12.4); men 48.6 (SD 12.5) and women 49.1 (SD 12.2). Age at first admission, expressed in years, was 25.1 (SD 8.8); men 23.7 (SD 7.8) and women 27.1 (SD 9.7), respectively. The total duration of admission, expressed in years, was 23.4 (SD 12.9), men 24.4 (SD 12.5) and women 22.0 (SD 13.4). Diagnoses according to DSM-IV Axis I as defined above were: schizophrenia 112 (66.7%), psychosis 9 (5.4%), affective disorder 27 (16.1%), other Axis I diagnosis 11 (6.6%) and no Axis I diagnosis 9 (5.4%).

### Association Analyses with SNPs

Six redundant SNPs owing to strong linkage disequilibrium (LD) (*Levwontin’s* D’ = 1, R-squared = 1) were removed (Table S1): rs879606, rs907094, rs3764353, rs3764352 in *PPP1R1B*, and rs4606 and rs1819741 in *RGS2*.

The following SNPs were excluded from analysis, due to deviation from HWE: all movement disorders – rs6280, as well as controls; TD - rs4795390; orofacial dyskinesia - rs4795390, rs1800497; limb truncal dyskinesia - rs1800497; bradykinesia - rs1799732, rs6311.

**Figure 1:**
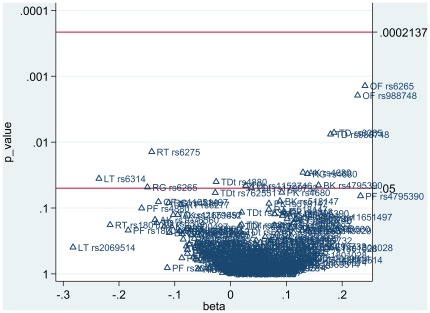
Smile plot summarizing set of multiple analyses after Simes correction for multiple testing. Corresponding p-values (on a reverse log scale against the corresponding parameter estimates). TD = tardive dyskinesia, OF = orofacial dyskinesia, LT = limb truncal dyskinesia, PK = parkinsonism, RT = rest tremor, RG = rigidity, BK = bradykinesia, AK = akathisia, TDt = tardive dystonia and PF = principal-factor.

The (multilevel) regression yielded significant coefficients, after adjustment for age, between tardive dyskinesia and rs6265 (B = 0.19, p = 0.0072) as well as rs988748 (B = 0.18, p = 0.0076); between orofacial dyskinesia and rs6265 (B = 0.24, p = 0.0014) as well as rs988748 (B = 0.23, p = 0.0019); and between limb truncal dyskinesia and rs6314 (B = −0.24, p = 0.0357). After adjustment for age and total antipsychotic DDD, associations were apparent between rest tremor and rs6275 (B = −0.14, p = 0.0140); between rigidity and rs6265 (B = −0.15, p = 0.0482) as well as rs4680 (B = 0.14, p = 0.0303); and between bradykinesia and rs4795390 (B = 0.16, p = 0.0451). Without adjustment, associations were apparent between akathisia and rs4680 (B = 0.13, p = 0.0289); between tardive dystonia and rs1799732 (B = 0.04, p = 0.0494), rs4880 (B = −0.03, p = 0.0399), as well as rs1152746 (B = 0.03, p = 0.0456). After Simes correction for multiple testing of the above mentioned analyses, the number of rejected p-values was zero, with a corrected overall critical p-value of 0.00021 (Figure 1).

Power calculations showed that our sample was insufficiently powered (0.05%) to identify the betas from our regressions, which were between −0.28 and 0.22.

## Discussion

In a population with chronic mental illness, various SNPs in 10 candidate genes (*PPP1R1B*, *BDNF*, *DRD3*, *DRD2*, *HTR2A*, *HTR2C*, *COMT*, *MnSOD*, *CYP1A2*, and *RGS2*) reached nominally significant (p≤0.05) associations with drug-induced movement disorder. However, after controlling for multiple testing, our findings suggest that these single nucleotide polymorphism (SNP) are not associated with a susceptibility to movement disorders.

Another reason for the inconclusive findings could be explained by the fact that in a naturalistic setting it is possible to evaluate the overall impact of pharmacogenetic signals in the presence of a host of real-life variables that can override pharmacogenetic variation. The fact we did not observe a significant association may also attest to the possibility that each gene makes a small contribution that is often diluted or overridden by environmental and clinical variations.

### Limitations

This study had limitations, for which we refer to our previous publications [21] (Bakker and colleagues, submitted) and additional limitations. First, as mentioned before, the relatively small sample size was the major limitation in this study. Still, the power in the current study may be increased as our patients had chronic mental illness, with a mean total duration of admission of 23.4 yrs (SD 12.9), which is a relatively long time for genetically susceptible patients to develop movement disorder. Also, we used continuous measures of movement disorder, which as a so-called intermediate quantitative trait is more informative about the underlying path in complex genetic diseases and thus generates more statistical power [32], [33]. In addition, we used repeated measures for continuous movement disorders, which may give a more stable phenotype, and thus more power.

Second, some authors may argue that association studies of movement disorders in patient with a psychotic disorder will produce non-significant results, as this model is inadequate since movement disorders may share risk alleles with schizophrenia [13]. However, many movement disorders and schizophrenia are complex diseases caused by multiple genetic and environmental factors, which are probably only partly shared, as (i) clinical heterogeneity in schizophrenia is clear, (ii) evidence of pathophysiological and etiological heterogeneity is accumulating [35], [36], and (iii) TD is a predictor for poor outcome of schizophrenia [37]. Hence, it can be hypothesized that patients with movement disorders represent a subgroup of schizophrenia and the above mentioned model is adequate.

Third, some authors may contend that medication is an important confounder, which should have been included in our analysis. However, a confounding mechanism is difficult to envisage, as choice of medication would need to be associated with an SNP and, independently thereof, with the movement disorder outcome. Nevertheless, medication may modify SNP-movement disorder outcomes and may be included in future analyses as an interaction term.

### Strengths

We refer to our previous publications [21] (Bakker and colleagues, submitted). The importance of repeated measures should be noted, as case definition of repeated measures, rather than a single cross-sectional measure, for continuous movement disorders better reflects the continuously fluctuating nature in time of movement disorders, and therefore may represent a more suitable standard in future research. To the best of our knowledge only few papers in the literature address this issue.

As the sample size of the current study is small with low power and previous studies show inconsistent results, definite conclusions cannot be made. Yet the question is how to interpret these results. In our opinion, the findings of weak genetic signals need to be replicated in larger study samples, preferably in longitudinal studies which take the fluctuating course of movement disorders and gene-environment interactions into account [38], [39]. Even though the current study is inconclusive, negative studies also ought to be reported as otherwise meta-analytic results in the future can be biased by positive studies that tend to be published more readily.

Various combinations of susceptibility genes may converge on synaptic processing in microcircuits, affecting a final common pathway of dysfunction and related symptoms, and secondary morphological alterations [40], [41]. However, despite growing evidence from genetic association studies, genetics only explains a minor part of schizophrenia, a fact which supports the importance of other interacting factors, such as environmental factors, which play important roles in schizophrenia [38]. Neuropsychiatric disorders may reflect the complex interplay of not only genetic factors, but first and foremost of epigenetic, stochastic, and non-genetic factors [42]. Consequently, at the moment it is too early to describe a genetic pathway of schizophrenia [38] or movement disorders.

An important development in human (pharmaco) genetics since 2005 is the possibility of genome-wide association studies (GWASs) [43] which have the advantage of a ‘hypothesis free’ and hence unbiased approach for examining new DNA variants which influence genetic susceptibility to many common diseases and can thus elucidate as yet unknown pathophysiological mechanisms.

After the choice of candidate genes in the current genetic association study was made, three GWASs of movement disorders were published: (i) the study by Inada e.a. [44] suggesting involvement of the GABA receptor signaling pathway in the development of therapy-resistant tardive dyskinesia, (ii) the study by Akelai e.a. [45] specifying EPF1, NOVA1, and FIGN as promising genes related to antipsychotic-induced parkinsonism, and (iii) the study by Åberg [46] determining an association between parkinsonism and a SNP in ZNF202, a transcriptional repressor controlling the major protein in myelin, PLP1, related both to Pelizaeus-Merzbacher disease with parkinsonism as symptom, and schizophrenia.

The Psychiatric GWAS Consortium (PGC) has suggested that in the near future larger GWAS samples will detect more variants of common susceptibility with smaller effect sizes and that meta-analyses of GWAS should find more conclusive evidence for genetic associations. Meanwhile, new potentially promising genetic techniques are being implemented such as epigenetics and whole-exome sequencing as an alternative study design. Rare variants detected by these next generation sequencing technologies may yield a stronger signal than GWAS approaches. In our view, the common variant common disease/phenotype approach is challenged including the area of pharmacogenetics. Rare variants warrant more attention in future studies. Also, gene-environment-wide interaction studies (GEWIS) approaches are being suggested [47]. It seems legitimate to conclude that these new techniques offer more effective genetic linkage and association studies.

There is a need for more participatory research designs, especially in naturalistic studies in personalized medicine including psychiatry. However, Lehoux and colleagues pose the following question to be answered: ‘what *value* does personalized medicine bring to health care?’ [48] This important question refers to the unique context of personalized medicine where economic, political and social issues come together.

In conclusion, the findings suggest that selected SNPs are not associated with a susceptibility to movement disorders. However, replication is needed in larger study samples, preferably in longitudinal studies which take the fluctuating course of movement disorders and gene-environment interactions into account. The use of intermediate phenotypes, for example, laboratory based phenotypes [42], or more accurate measures of movement disorders, for example instrument measurement of lingual force variability as proposed by Koning and colleagues [49], which may represent a powerful alternative since instrument measurement detects subclinical movement disorders and is highly reliable. Moreover, (pharmaco) genetic studies may help elucidate common pathways in the development of movement disorders. With this information, an alternative World Health Organization Model List of Essential Medicines may be one that lists the ‘minimal essential biomarkers’ required for optimal pharmacotherapy [50]. However, on balance, our findings should be set in the context of interactions with both other genetic susceptibility loci and environmental factors, and, as rightly stated by Faraone and colleagues [51] “any conclusion about the role of genes and environment must rely not on a single study or class of study but on the converging evidence provided by a variety of research paradigms.”

Future research on movement disorders may be served by the inclusion of all four movement disorder, as performed in the current study, since they may represent pleiotropic effects from (partly) shared genetic factors [52].

## Supporting Information

Text S1 **Supporting information about the 10 candidate genes.**
(DOC)

Table S1 **Selected 31 SNPs for multilevel regression of continuous movement disorders.**
(DOC)
