# Supplementary material for: Candidate Gene-Based Association Study of Antipsychotic-Induced Movement Disorders in Long-Stay Psychiatric Patients: A Prospective Study
Source: PLoS One. 2012 May 15;7(5):e36561. doi: 10.1371/journal.pone.0036561 (PMC3352907; doi:10.1371/journal.pone.0036561)
Supplement: Text S1 — Supporting information about the 10 candidate genes. (DOC) [file pone.0036561.s001.doc]

Supporting Text S1

## Genes

A systematic literature review was conducted of the literature published between 1976 and August 2011, with the help of Medline, EMBASE and PsychINFO using key words (genetic) polymorphism(s), tardive dyskinesia, extrapyramidal (syndrome/disorder), *drug-induced,* *antipsychotic(s), adverse effect*/event, schizophrenia. In addition, all relevant references cited in these articles were also retrieved.

***S1.1 Tardive dyskinesia***

The dopaminergic and serotonergic systems of neurotransmission have been implicated in the development of movement disorders.

Genes involved in dopaminergic signaling, possibly associated with the development of TD, include those coding for: (i) Dopamine 3 receptor (*DRD3*), with evidence from meta-analyses for an association between Ser9Gly (rs6280) and TD [1,2], but no or little evidence in a recent meta-analysis [3], confirming the observation of progressive reduction of meta-analytic effects over time in genetic studies [4,5]; (ii) Dopamine 2 receptor (*DRD2*), with evidence from two meta-analyses for an association between Taq1A (rs1800497) and TD [6,7]. In a recent study with Korean patients, 5 SNPs in *DRD2* (-141Ins/Del/Taq1D/NcoI/Ser311Cys/Taq1A) showed no association with TD and TD severity, or the haplotype of these 5 SNPs with TD [8]. Another recent study found evidence for an association between -141Ins/Del (rs1799732) and TD [9]; (iii) Brain-derived neurotrophic factor (*BDNF*), albeit to date without reported association between Val66Met (rs6265) and TD [10,11]. Furthermore, Xu and colleagues [12] showed that the (GT)_n_ repeat polymorphism of the *BDNF* gene may be an independent contributor to chlorpromazine-induced TD, akathisia and parkinsonism; and (iv) Catechol-O-methyltransferase (*COMT*), with evidence from one meta-analysis for an association between Val158Met (rs4680) and TD, where the Val-variant had a risk increasing effect on TD [7]. A study reported that one of six SNPs (rs165599) in the *COMT* gene may be associated with TD in men, and a sex-stratified meta-analysis showed a significant association between Val158Met (rs4680) and TD, where the ValVal-genotype had a risk increasing effect on TD using the fixed-effect model unadjusted for sex, and in females using the random effect model [13].

Serotonergic genes studied in TD include those coding for: (i) Serotonin 2A receptor (*HTR2A)*, with evidence for an association of TD with T102C (rs6313) detected by Lerer and colleagues [14], also after adjustment for age, by pooled meta-analysis, and with the T102C-His452Tyr haplotype, while other studies failed to find significant effects for rs6313 [15,16]. For Hist452Tyr (rs6314), in *HTR2A*, no association with TD was detected [14], nor for A-1438G (rs6311) [15,16]. rs6311 was significantly associated with TD in a Turkish population, however only when cumulative antipsychotic intake was considered [17]; (ii) Serotonin 2C receptor (*HTR2C)*, with evidence for an association between -697G/C (rs518147) and TD, but not for -759-T/C (rs3813929) or the haplotype of both [18]. For Cys23Ser (rs6318) an age-related effect with AIMS was found [19]. Another study did not detect differences in allele frequencies of -997A, -759T or -697C between groups of TD, non-TD and controls, whereas the 23Ser allele was significantly higher in patients with movement disorders, with a similar trend using haplotypes of these 4 SNPs [20]. Furthermore, both -697G/C and -759-T/C polymorphisms were associated with the emergence of TD [21].

Findings in a recent study of TD with 128 candidate genes (amongst them dopamine, serotonin) did not support significant results for either novel or prior associations from the literature [22]. Similarly, in a recent study, no association was found between serotonergic genes (amongst others *HTR2A, HTR2C*) and movement disorders [23].

Another study showed that limb truncal, but not orofaciolingual, TD was associated with Ser9Gly (*DRD3*) and Cys23Ser (*HTR2C*) in a Russian population, whereas neither subform of TD with associated with A-1438G (*HTR2A*) [24].

A polymorphism in intron 1 of *CYP1A2* (-163C>A; *CYP1A2**1F allele; rs762551) appears to affect the inducibility of CYP1A2 by smoking [25,26]. The *CYP1A2**1C allele (-3860G>A; rs2069514) also results in a lower activity in smokers [27]. A meta-analysis did not find an association between both SNPs in *CYP1A2* and TD [7]. Furthermore, Tiwari and colleagues [28] did not find significant results between TD and different SNPs in *CYP1A2*, nor did Boke and colleagues [17] for rs762551 in a Turkish population.

Complementary to the ‘dopamine supersensitivity hypothesis’ on TD**,** the hypothesis of neuronal degeneration owing to toxic effects of free radicals has been proposed, and free radical scavenging enzymes like manganese super oxide dismutase (*MnSOD*) have been investigated [29]. A meta-analysis showed genetic association with TD in Ala-9Val in *MnSOD* [7]. However, a more recent study with subsequent meta-analysis did not find significant results between *MnSOD* Ala-9Val (rs4880) and TD [30] confirming the observation of progressive reduction of meta-analytic effects over time in genetic studies [4,5]. A significant association between Ala-9Val and severity of TD, but not TD as dichotomous outcome, has been reported [31]. Another study by al Hadithy and colleagues [32] found a significant association between Ala-9Val and orofaciolingual TD in a Russian population. A recent study reported no evidence for an association between Ala-9Val and TD in Han Chinese [33].

A relatively new and interesting candidate gene is *PPP1R1B*, which encodes phosphatase 1, regulatory (inhibitor) subunit 1B (PPP1R1B), also known as dopamine and cAMP regulated phosphoprotein of 32 kDa (DARPP-32), an important regulatory molecule in both the dopaminergic [34] and glutamatergic signaling pathways, which is selectively expressed in neostriatal spiny neurons [35]. Deficit of DARPP-32 in striatonigral neurons decreased basal and cocaine-induced locomotion and stopped L-DOPA induced dyskinetic behaviors. On the other hand, the deficit of DARPP-32 in striatopallidal neurons produced a strong increase in locomotor activity and a strongly reduced cataleptic reaction to haloperidol [36]. To date, only one study examined TD and PPP1R1B, however without result [37].

More details on this topic can be found in the recent extensive review by Lee and Kang [38].

***S1.2. Parkinsonism***

The pharmacological explanation of drug-induced parkinsonism (DIP) is antagonism of the nigrostriatal dopamine D2 receptor [39,40]. One study in an African-Caribbean population found a significant association between the -141Ins/Del polymorphism in *DRD2* and rigidity in males, as well as between the Cys23Ser polymorphism and bradykinesia [41].

The regulator of G-protein signaling 2 (*RGS2*) may play a role in DIP, as it is involved in *HTR2A* and muscarinic receptor (M1 and M3) signaling, and antagonism of these receptors results in a decrease of DIP [42].

Greenbaum and colleagues [43] reported a significant association between rs4606 in *RGS2* and DIP in Jewish participants, which was confirmed by a replication study in an African-American subsample from a mixed population with whites [44]. Another study did not confirm this association in an African-Caribbean population [42], which may be explained by the lower use of atypical antipsychotics in the latter study, most of these agents being *HTR2A* antagonists and muscarinic receptor (M1 and M3) antagonists [42]. A recent study in a Japanese population found an association between rs4606 and parkinsonism, which disappeared when covariates were considered [45].

***S1.3 Akathisia***

An association between the Ser9Gly polymorphism in *DRD3* and the risk to develop akathisia has been reported [46]. A recent study found evidence for an association between Taq1D (rs1800498) and akathisia [9].

***S1.4 Tardive dystonia***

Genes coding for CYP2D6, DRD2 and DRD3 did not show an association with tardive dystonia [47].

***S1.5 Gene-gene interactions***

Combined pharmacokinetic and pharmacogenetic aspects of antipsychotics may help finding subpopulations liable to develop TD [48–50]. Segman and colleagues [51] suggested that the variance of orofacial tardive dyskinesia (OFD) explained by *DRD3* and *HTR2C* may be as high as 20.9 %. Carriership of both risk-alleles explained 4.2% en 4.7%, respectively. Carriers of the risk *DRD3*-Gly allele and the risk-genotype A2-A2 of *CYP17* displayed the highest rate of orofacial, distal and incapacitation scores on the AIMS [52]. *DRD3*- and *CYP1A2*-genotypes together accounted for most of the variance of the severest form of TD, the explained variance being > 50% [53]. In one study, in a Chinese Han population, Ser9Gly in *DRD3* was not associated with TD, however in combination with Ala-9Val in *MnSOD* it was [54].

In an African-Caribbean population, evidence for association was reported between the AIMS and (i) Ser9Gly (*DRD3*) in females, (ii) Ser9Gly with Cys23Ser (*HTR2C*) or A-1438G (*HTR2A*) in males, (iii) Cys23Ser (*HTR2C*) with A-1438G (*HTR2A*) in males [55].

One study found evidence for association between a haplotype containing rs3732782, rs905568, and rs7620754 in the 5' region of *DRD3* on the one hand, and both TD and AIMS on the other, as well as evidence for interaction between *BDNF* (rs11030104) and *DRD3* polymorphisms (rs2087017, rs167770, rs7633291 and rs9825563) and the AIMS, albeit not for BNDF genetic markers in isolation [56].

One study showed a significant association between *BDNF* Val66Met and AIMS orofacial scores, and a trend of higher AIMS total and limb-trunk scores. However, AIMS scores and the combination of *DRD3* ser9gly with *BDNF* val66met were not associated. Furthermore, TD was not associated with *DRD3* or *BDNF* [57].

One study found a significant combined association between val66met in *BDNF* and -50T/C in GSK-3beta polymorphisms on the one hand and TD on the other, but not with any of the polymorphisms separately [58].

# References

1. Lerer B, Segman RH, Fangerau H, Daly AK, Basile VS, et al. (2002) Pharmacogenetics of tardive dyskinesia: combined analysis of 780 patients supports association with dopamine D3 receptor gene Ser9Gly polymorphism. Neuropsychopharmacology 27: 105-19.

2. Bakker PR, van Harten PN, van Os J (2006) Antipsychotic-induced tardive dyskinesia and the Ser9Gly polymorphism in the DRD3 gene: a meta analysis. Schizophr Res 83: 185-192.

3. Tsai HT, North KE, West SL, Poole C (2010) The DRD3 rs6280 polymorphism and prevalence of tardive dyskinesia: a meta-analysis. Am J Med Genet B Neuropsychiatr Genet 153B: 57-66.

4. Lencz T, Malhotra AK (2009) Pharmacogenetics of antipsychotic-induced side effects. Dialogues Clin Neurosci 11: 405-415.

5. Xiao R, Boehnke M (2009) Quantifying and correcting for the winner's curse in genetic association studies. Genet Epidemiol 33: 453-462.

6. Zai CC, De Luca V, Hwang RW, Voineskos A, Muller DJ, et al. (2007) Meta-analysis of two dopamine D2 receptor gene polymorphisms with tardive dyskinesia in schizophrenia patients. Mol Psychiatry 12: 794-795.

7. Bakker PR, van Harten PN, van Os J (2008) Antipsychotic-induced tardive dyskinesia and polymorphic variations in COMT, DRD2, CYP1A2 and MnSOD genes: a meta-analysis of pharmacogenetic interactions. Mol Psychiatry 13: 544-556.

8. Park YM, Kang SG, Choi JE, Kim YK, Kim SH, et al. (2011) No Evidence for an Association between Dopamine D2 Receptor Polymorphisms and Tardive Dyskinesia in Korean Schizophrenia Patients. Psychiatry Investig 8: 49-54. 10.4306/pi.2011.8.1.49 [doi].

9. Koning JP, Vehof J, Burger H, Wilffert B, Al Hadithy A, et al. (2011) Association of two DRD2 gene polymorphisms with acute and tardive antipsychotic-induced movement disorders in young Caucasian patients. Psychopharmacology (Berl) . 10.1007/s00213-011-2394-1 [doi].

10. Wang Y, Wang JD, Wu HR, Zhang BS, Fang H, et al. (2010) The Val66Met polymorphism of the brain-derived neurotrophic factor gene is not associated with risk for schizophrenia and tardive dyskinesia in Han Chinese population. Schizophr Res 120: 240-242.

11. Kang SG, Choi JE, An H, Lim SW, Lee HJ, et al. (2008) No association between the brain-derived neurotrophic factor gene Val66Met polymorphism and tardive dyskinesia in schizophrenic patients. Prog Neuropsychopharmacol Biol Psychiatry 32: 1545-1548.

12. Xu MQ, St Clair D, Feng GY, Lin ZG, He G, et al. (2008) BDNF gene is a genetic risk factor for schizophrenia and is related to the chlorpromazine-induced extrapyramidal syndrome in the Chinese population. Pharmacogenet Genomics 18: 449-457.

13. Zai CC, Tiwari AK, Muller DJ, De Luca V, Shinkai T, et al. (2010) The catechol-O-methyl-transferase gene in tardive dyskinesia. World J Biol Psychiatry 11: 803-812.

14. Lerer B, Segman RH, Tan EC, Basile VS, Cavallaro R, et al. (2005) Combined analysis of 635 patients confirms an age-related association of the serotonin 2A receptor gene with tardive dyskinesia and specificity for the non-orofacial subtype. Int J Neuropsychopharmacol 8: 411-425.

15. Herken H, Erdal ME, Boke O, Savas HA (2003) Tardive dyskinesia is not associated with the polymorphisms of 5-HT2A receptor gene, serotonin transporter gene and catechol-o-methyltransferase gene. Eur Psychiatry 18: 77-81.

16. Deshpande SN, Varma PG, Semwal P, Rao AR, Bhatia T, et al. (2005) II. Serotonin receptor gene polymorphisms and their association with tardive dyskinesia among schizophrenia patients from North India. Psychiatr Genet 15: 157-158.

17. Boke O, Gunes S, Kara N, Aker S, Sahin AR, et al. (2007) Association of serotonin 2A receptor and lack of association of CYP1A2 gene polymorphism with tardive dyskinesia in a Turkish population. DNA Cell Biol 26: 527-531.

18. Zhang ZJ, Zhang XB, Sha WW, Zhang XB, Reynolds GP (2002) Association of a polymorphism in the promoter region of the serotonin 5-HT2C receptor gene with tardive dyskinesia in patients with schizophrenia. Mol Psychiatry 7: 670-671.

19. Segman RH, Lerer B (2002) Age and the relationship of dopamine D3, serotonin 2C and serotonin 2A receptor genes to abnormal involuntary movements in chronic schizophrenia. Mol Psychiatry 7: 137-139.

20. Gunes A, Dahl ML, Spina E, Scordo MG (2008) Further evidence for the association between 5-HT2C receptor gene polymorphisms and extrapyramidal side effects in male schizophrenic patients. Eur J Clin Pharmacol 64: 477-482.

21. Rizos EN, Siafakas N, Katsantoni E, Lazou V, Sakellaropoulos K, et al. (2009) Association of the dopamine D3 receptor Ser9Gly and of the serotonin 2C receptor gene polymorphisms with tardive dyskinesia in Greeks with chronic schizophrenic disorder. Psychiatr Genet 19: 106-107.

22. Tsai HT, Caroff SN, Miller DD, McEvoy J, Lieberman JA, et al. (2010) A candidate gene study of Tardive dyskinesia in the CATIE schizophrenia trial. Am J Med Genet B Neuropsychiatr Genet 153B: 336-340.

23. Al-Janabi I, Arranz MJ, Blakemore AI, Saiz PA, Susce MT, et al. (2009) Association study of serotonergic gene variants with antipsychotic-induced adverse reactions. Psychiatr Genet 19: 305-311.

24. Al Hadithy AF, Ivanova SA, Pechlivanoglou P, Semke A, Fedorenko O, et al. (2009) Tardive dyskinesia and DRD3, HTR2A and HTR2C gene polymorphisms in Russian psychiatric inpatients from Siberia. Prog Neuropsychopharmacol Biol Psychiatry 33: 475-481.

25. MacLeod SL, Tang YM, Yokoi T, Kamataki T, Doublin S, et al. (1998) The role of a recently discovered genetic polymorphism in the regulation of the human CYP1A2 gene. Proc Am Assoc Cancer Res 39: 396.

26. Sachse C, Brockmoller J, Bauer S, Roots I (1999) Functional significance of a C-->A polymorphism in intron 1 of the cytochrome P450 CYP1A2 gene tested with caffeine. Br J Clin Pharmacol 47: 445-449.

27. Nakajima M, Yokoi T, Mizutani M, Kinoshita M, Funayama M, et al. (1999) Genetic polymorphism in the 5'-flanking region of human CYP1A2 gene: effect on the CYP1A2 inducibility in humans. J Biochem (Tokyo) 125: 803-808.

28. Tiwari AK, Deshpande SN, Lerer B, Nimgaonkar VL, Thelma BK (2007) Genetic susceptibility to Tardive Dyskinesia in chronic schizophrenia subjects: V. Association of CYP1A2 1545 C>T polymorphism. Pharmacogenomics J 7: 305-311.

29. Tsai G, Goff DC, Chang RW, Flood J, Baer L, et al. (1998) Markers of glutamatergic neurotransmission and oxidative stress associated with tardive dyskinesia. Am J Psychiatry 155: 1207-1213.

30. Zai CC, Tiwari AK, Basile V, De Luca V, Muller DJ, et al. (2010) Oxidative stress in tardive dyskinesia: genetic association study and meta-analysis of NADPH quinine oxidoreductase 1 (NQO1) and Superoxide dismutase 2 (SOD2, MnSOD) genes. Prog Neuropsychopharmacol Biol Psychiatry 34: 50-56.

31. Kang SG, Choi JE, An H, Park YM, Lee HJ, et al. (2008) Manganese superoxide dismutase gene Ala-9Val polymorphism might be related to the severity of abnormal involuntary movements in Korean schizophrenic patients. Prog Neuropsychopharmacol Biol Psychiatry 32: 1844-1847.

32. Al Hadithy AF, Ivanova SA, Pechlivanoglou P, Wilffert B, Semke A, et al. (2010) Missense polymorphisms in three oxidative-stress enzymes (GSTP1, SOD2, and GPX1) and dyskinesias in Russian psychiatric inpatients from Siberia. Hum Psychopharmacol 25: 84-91.

33. Liu H, Wang C, Chen PH, Zhang BS, Zheng YL, et al. (2010) Association of the manganese superoxide dismutase gene Ala-9Val polymorphism with clinical phenotypes and tardive dyskinesia in schizophrenic patients. Prog Neuropsychopharmacol Biol Psychiatry 34: 692-696. S0278-5846(10)00119-3 [pii];10.1016/j.pnpbp.2010.03.026 [doi].

34. Yoshimi A, Takahashi N, Saito S, Ito Y, Aleksic B, et al. (2008) Genetic analysis of the gene coding for DARPP-32 (PPP1R1B) in Japanese patients with schizophrenia or bipolar disorder. Schizophr Res 100: 334-341.

35. Hu JX, Yu L, Shi YY, Zhao XZ, Meng JW, et al. (2007) An association study between PPP1R1B gene and schizophrenia in the Chinese population. Prog Neuropsychopharmacol Biol Psychiatry 31: 1303-1306.

36. Bateup HS, Santini E, Shen W, Birnbaum S, Valjent E, et al. (2010) Distinct subclasses of medium spiny neurons differentially regulate striatal motor behaviors. Proc Natl Acad Sci U S A 107: 14845-14850.

37. Tiwari AK, Souza RP, Zai CC, Mueller DJ, Remington G, et al (2009) Lack of association of the Dopamine and cAMP-regulated phosphoprotein (MW=32 kDa; DARPP-32; PPP1R1B) gene to Tardive Dyskinesia in chronic schizophrenia patients (SOBP, Vancouver, May 14-16, 2009).

38. Lee HJ, Kang SG (2011) Genetics of tardive dyskinesia. Int Rev Neurobiol 98: 231-264. B978-0-12-381328-2.00010-9 [pii];10.1016/B978-0-12-381328-2.00010-9 [doi].

39. Reynolds GP (2004) Receptor mechanisms in the treatment of schizophrenia. J Psychopharmacol 18: 340-345.

40. Sachdev PS (2005) Neuroleptic-induced movement disorders: an overview. Psychiatr Clin North Am 28: 255-74, x.

41. Al Hadithy AF, Wilffert B, Stewart RE, Looman NM, Bruggeman R, et al. (2008) Pharmacogenetics of parkinsonism, rigidity, rest tremor, and bradykinesia in African-Caribbean inpatients: differences in association with dopamine and serotonin receptors. Am J Med Genet B Neuropsychiatr Genet 147B: 890-897.

42. Al Hadithy AF, Wilffert B, Bruggeman R, Stewart RE, Brouwers JR, et al. (2009) Lack of association between antipsychotic-induced Parkinsonism or its subsymptoms and rs4606 SNP of RGS2 gene in African-Caribbeans and the possible role of the medication: The Curacao extrapyramidal syndromes study X. Hum Psychopharmacol 24: 123-128.

43. Greenbaum L, Strous RD, Kanyas K, Merbl Y, Horowitz A, et al. (2007) Association of the RGS2 gene with extrapyramidal symptoms induced by treatment with antipsychotic medication. Pharmacogenet Genomics 17: 519-528.

44. Greenbaum L, Smith RC, Rigbi A, Strous R, Teltsh O, et al. (2009) Further evidence for association of the RGS2 gene with antipsychotic-induced parkinsonism: protective role of a functional polymorphism in the 3'-untranslated region. Pharmacogenomics J 9: 103-110.

45. Higa M, Ohnuma T, Maeshima H, Hatano T, Hanzawa R, et al. (2010) Association analysis between functional polymorphism of the rs4606 SNP in the RGS2 gene and antipsychotic-induced Parkinsonism in Japanese patients with schizophrenia: results from the Juntendo University Schizophrenia Projects (JUSP). Neurosci Lett 469: 55-59.

46. Eichhammer P, Albus M, Borrmann-Hassenbach M, Schoeler A, Putzhammer A, et al. (2000) Association of dopamine D3-receptor gene variants with neuroleptic induced akathisia in schizophrenic patients: a generalization of Steen's study on DRD3 and tardive dyskinesia. Am J Med Genet 96: 187-91.

47. Mihara K, Kondo T, Higuchi H, Takahashi H, Yoshida K, et al. (2002) Tardive dystonia and genetic polymorphisms of cytochrome P4502D6 and dopamine D2 and D3 receptors: a preliminary finding. Am J Med Genet 114: 693-695.

48. Ozdemir V, Aklillu E, Mee S, Bertilsson L, Albers LJ, et al. (2006) Pharmacogenetics for off-patent antipsychotics: reframing the risk for tardive dyskinesia and access to essential medicines. Expert Opin Pharmacother 7: 119-133.

49. Ozdemir V, Basile VS, Masellis M, Kennedy JL (2001) Pharmacogenetic assessment of antipsychotic-induced movement disorders: contribution of the dopamine D3 receptor and cytochrome P450 1A2 genes. J Biochem Biophys Methods 47: 151-157.

50. Faraone SV, Tsuang MT, Tsuang DW (1999) Genetic of mental disorders. New York: The Guilford Press.

51. Segman RH, Heresco-Levy U, Finkel B, Inbar R, Neeman T, et al. (2000) Association between the serotonin 2C receptor gene and tardive dyskinesia in chronic schizophrenia: additive contribution of 5-HT2Cser and DRD3gly alleles to susceptibility. Psychopharmacology (Berl) 152: 408-413.

52. Segman RH, Heresco-Levy U, Yakir A, Goltser T, Strous R, et al. (2002) Interactive effect of cytochrome P450 17alpha-hydroxylase and dopamine D3 receptor gene polymorphisms on abnormal involuntary movements in chronic schizophrenia. Biol Psychiatry 51: 261-263.

53. Basile VS, Masellis M, Potkin SG, Kennedy JL (2002) Pharmacogenomics in schizophrenia: the quest for individualized therapy. Hum Mol Genet 11: 2517-30.

54. Zhang ZJ, Zhang XB, Hou G, Yao H, Reynolds GP (2003) Interaction between polymorphisms of the dopamine D3 receptor and manganese superoxide dismutase genes in susceptibility to tardive dyskinesia. Psychiatr Genet 13: 187-192.

55. Wilffert B, Al Hadithy AF, Sing VJ, Matroos G, Hoek HW, et al. (2009) The role of dopamine D3, 5-HT2A and 5-HT2C receptor variants as pharmacogenetic determinants in tardive dyskinesia in African-Caribbean patients under chronic antipsychotic treatment: Curacao extrapyramidal syndromes study IX. J Psychopharmacol 23: 652-659.

56. Zai CC, Tiwari AK, De Luca V, Muller DJ, Bulgin N, et al. (2009) Genetic study of BDNF, DRD3, and their interaction in tardive dyskinesia. Eur Neuropsychopharmacol 19: 317-328.

57. Liou YJ, Liao DL, Chen JY, Wang YC, Lin CC, et al. (2004) Association analysis of the dopamine D3 receptor gene ser9gly and brain-derived neurotrophic factor gene val66met polymorphisms with antipsychotic-induced persistent tardive dyskinesia and clinical expression in Chinese schizophrenic patients. Neuromolecular Med 5: 243-251.

58. Park SW, Lee JG, Kong BG, Lee SJ, Lee CH, et al. (2009) Genetic association of BDNF val66met and GSK-3beta-50T/C polymorphisms with tardive dyskinesia. Psychiatry Clin Neurosci 63: 433-439.
