# Supplementary material for: Candidate Gene-Based Association Study of Antipsychotic-Induced Movement Disorders in Long-Stay Psychiatric Patients: A Prospective Study
Source: PLoS One. 2012 May 15;7(5):e36561. doi: 10.1371/journal.pone.0036561 (PMC3352907; doi:10.1371/journal.pone.0036561)
Supplement: Table S1 — Selected 31 SNPs for multilevel regression of continuous movement disorders. (DOC) [file pone.0036561.s002.doc]

**Table S1.** *Selected 31 SNPs for multilevel regression of continuous movement disorders*

|  | **SNP** | **Chromosome Position** | **Alleles Public** | **LD (r^2^)^a^** | **TD^b^** |  | **OF^b^** |  | **LT^b^** |  | **PK^b^** |  | **RT^b^** |  | **RG^b^** |  | **BK^b^** |  | **AK^b^** |  | **TDt^b^** |  | **PF^b^** |  |
| --- | --- | --- | --- | --- | --- | --- | --- | --- | --- | --- | --- | --- | --- | --- | --- | --- | --- | --- | --- | --- | --- | --- | --- | --- |
|  |  |  |  |  |  |  |  |  |  |  |  |  |  |  |  |  |  |  |  |  |  |  |  |  |
|  |  |  | *Major/Minor* |  | *Beta* | *p_value* | *Beta* | *p_value* | *Beta* | *p_value* | *Beta* | *p_value* | *Beta* | *p_value* | *Beta* | *p_value* | *Beta* | *p_value* | *Beta* | *p_value* | *Beta* | *p_value* | *Beta* | *p_value* |
| ***PPP1R1B*** |  | chr17:37783179-37792875 |  |  |  |  |  |  |  |  |  |  |  |  |  |  |  |  |  |  |  |  |  |  |
|  | rs4795390 | [chr17:37781360-37781860](http://genome.ucsc.edu/cgi-bin/hgTracks?position=chr17:37781360-37781860&hgsid=247045277&snp135Common=pack&hgFind.matches=rs4795390,) | CG | 1.00 |  |  |  |  | -0.08 | 0.3794 | 0.10 | 0.1196 | 0.03 | 0.6203 | 0.01 | 0.8827 | 0.16 | 0.0451 | 0.00 | 0.9535 | -0.00 | 0.9892 | 0.23 | 0.0657 |
|  | rs879606 | [chr17:37781599-37782099](http://genome.ucsc.edu/cgi-bin/hgTracks?position=chr17:37781599-37782099&hgsid=247045277&snp135Common=pack&hgFind.matches=rs879606,) | GA | 0.62 |  |  |  |  |  |  |  |  |  |  |  |  |  |  |  |  |  |  |  |  |
|  | rs11651497 | [chr17:37789748-37790248](http://genome.ucsc.edu/cgi-bin/hgTracks?position=chr17:37789748-37790248&hgsid=247045277&snp135Common=pack&hgFind.matches=rs11651497,) | CT | 1.00 | -0.10 | 0.1269 | -0.13 | 0.0807 | -0.07 | 0.3508 | 0.05 | 0.3345 | 0.04 | 0.5410 | -0.04 | 0.5646 | 0.09 | 0.1877 | 0.00 | 0.9506 | -0.01 | 0.6948 | 0.17 | 0.1361 |
|  | rs907094 | [chr17:37790121-37790621](http://genome.ucsc.edu/cgi-bin/hgTracks?position=chr17:37790121-37790621&hgsid=247045277&snp135Common=pack&hgFind.matches=rs907094,) | TC | 1.00 |  |  |  |  |  |  |  |  |  |  |  |  |  |  |  |  |  |  |  |  |
|  | rs3764353 | [chr17:37790531-37791031](http://genome.ucsc.edu/cgi-bin/hgTracks?position=chr17:37790531-37791031&hgsid=247045277&snp135Common=pack&hgFind.matches=rs3764353,) | GA | 1.00 |  |  |  |  |  |  |  |  |  |  |  |  |  |  |  |  |  |  |  |  |
|  | rs3764352 | [chr17:37790689-37791189](http://genome.ucsc.edu/cgi-bin/hgTracks?position=chr17:37790689-37791189&hgsid=247045277&snp135Common=pack&hgFind.matches=rs3764352,) | AG |  |  |  |  |  |  |  |  |  |  |  |  |  |  |  |  |  |  |  |  |  |
| ***BDNF*** |  | chr11:27528399-27699348 |  |  |  |  |  |  |  |  |  |  |  |  |  |  |  |  |  |  |  |  |  |  |
|  | rs6265 | [chr11:27679666-27680166](http://genome.ucsc.edu/cgi-bin/hgTracks?position=chr11:27679666-27680166&hgsid=247045277&gwasCatalog=pack&hgFind.matches=rs6265,) | GA | 0.80 | 0.19 | 0.0072 | 0.24 | 0.0014 | 0.11 | 0.1681 | -0.07 | 0.2125 | -0.08 | 0.2055 | -0.15 | 0.0482 | -0.05 | 0.5006 | 0.09 | 0.2172 | 0.02 | 0.2294 | -0.06 | 0.5949 |
|  | rs988748 | [chr11:27724495-27724995](http://genome.ucsc.edu/cgi-bin/hgTracks?position=chr11:27724495-27724995&hgsid=247045277&snp135Common=pack&hgFind.matches=rs988748,) | CG |  | 0.18 | 0.0076 | 0.23 | 0.0019 | 0.12 | 0.1510 | -0.02 | 0.7069 | -0.04 | 0.4580 | -0.05 | 0.4591 | -0.00 | 0.9867 | 0.10 | 0.1450 | 0.02 | 0.1786 | -0.03 | 0.7992 |
| ***DRD3*** |  | chr3:113847557-113897899 |  |  |  |  |  |  |  |  |  |  |  |  |  |  |  |  |  |  |  |  |  |  |
|  | rs6280 | [chr3:113890565-113891065](http://genome.ucsc.edu/cgi-bin/hgTracks?position=chr3:113890565-113891065&hgsid=247045277&snp135Common=pack&hgFind.matches=rs6280,) | TC | - |  |  |  |  |  |  |  |  |  |  |  |  |  |  |  |  |  |  |  |  |
| ***DRD2*** |  | chr11:113280318-113346001 |  |  |  |  |  |  |  |  |  |  |  |  |  |  |  |  |  |  |  |  |  |  |
|  | rs1800497 | [chr11:113270578-113271078](http://genome.ucsc.edu/cgi-bin/hgTracks?position=chr11:113270578-113271078&hgsid=247045277&snp135Common=pack&hgFind.matches=rs1800497,) | A2A1(=CT) | 0.18 | 0.04 | 0.6700 |  |  |  |  | -0.04 | 0.5653 | 0.06 | 0.4732 | 0.02 | 0.8531 | -0.11 | 0.2367 | -0.12 | 0.1900 | -0.01 | 0.6755 | -0.18 | 0.2176 |
|  | rs6277 | [chr11:113283209-113283709](http://genome.ucsc.edu/cgi-bin/hgTracks?position=chr11:113283209-113283709&hgsid=247045277&snp135Common=pack&hgFind.matches=rs6277,) | TC | 0.57 | -0.01 | 0.9222 | -0.02 | 0.7872 | 0.02 | 0.8119 | -0.05 | 0.3217 | -0.09 | 0.0890 | -0.03 | 0.6671 | -0.03 | 0.6154 | -0.06 | 0.3377 | 0.00 | 0.8052 | 0.00 | 0.9660 |
|  | rs6275 | [chr11:113283227-113283727](http://genome.ucsc.edu/cgi-bin/hgTracks?position=chr11:113283227-113283727&hgsid=247045277&snp135Common=pack&hgFind.matches=rs6275,) | CT | 0.07 | -0.05 | 0.4662 | -0.05 | 0.5429 | -0.05 | 0.5266 | -0.05 | 0.3497 | -0.14 | 0.0140 | -0.06 | 0.3809 | 0.00 | 0.9981 | -0.00 | 0.9688 | 0.01 | 0.6949 | 0.05 | 0.6504 |
|  | rs1801028 | [chr11:113283234-113283734](http://genome.ucsc.edu/cgi-bin/hgTracks?position=chr11:113283234-113283734&hgsid=247045277&snp135Common=pack&hgFind.matches=rs1801028,) | SerCys(=CG) | 0.00 | 0.15 | 0.4085 | 0.13 | 0.5163 | 0.18 | 0.3967 | -0.04 | 0.8049 | -0.22 | 0.1802 | -0.04 | 0.8532 | 0.06 | 0.7399 | 0.05 | 0.8034 | -0.01 | 0.7256 | -0.06 | 0.8451 |
|  | rs1076560 | [chr11:113283438-113283938](http://genome.ucsc.edu/cgi-bin/hgTracks?position=chr11:113283438-113283938&hgsid=247045277&snp135Common=pack&hgFind.matches=rs1076560,) | CA | 0.00 | 0.08 | 0.3870 | 0.04 | 0.6712 | 0.14 | 0.2069 | -0.02 | 0.8119 | 0.06 | 0.4611 | 0.06 | 0.5787 | -0.08 | 0.4292 | -0.14 | 0.1507 | -0.00 | 0.8756 | -0.09 | 0.5734 |
|  | rs1799732 | [chr11:113346003-113346502](http://genome.ucsc.edu/cgi-bin/hgTracks?position=chr11:113346003-113346502&hgsid=247045277&snp135=pack&hgFind.matches=rs1799732,) | CDel |  | 0.00 | 0.9701 | -0.08 | 0.4274 | 0.11 | 0.3099 | 0.02 | 0.7412 | -0.02 | 0.7729 | 0.02 | 0.8459 |  |  | 0.03 | 0.7139 | 0.04 | 0.0494 | 0.13 | 0.3809 |
| ***HTR2A*** |  | chr13:47407513-47470369 |  |  |  |  |  |  |  |  |  |  |  |  |  |  |  |  |  |  |  |  |  |  |
|  | rs6314 | [chr13:47408784-47409284](http://genome.ucsc.edu/cgi-bin/hgTracks?position=chr13:47408784-47409284&hgsid=247045277&snp135Common=pack&hgFind.matches=rs6314,) | HisTyr=CT | 0.01 | -0.13 | 0.1680 | -0.05 | 0.6390 | -0.24 | 0.0357 | 0.07 | 0.4092 | 0.02 | 0.8300 | 0.13 | 0.2186 | 0.07 | 0.5000 | -0.04 | 0.6902 | 0.01 | 0.8044 | 0.04 | 0.8179 |
|  | rs6313 | [chr13:47469690-47470190](http://genome.ucsc.edu/cgi-bin/hgTracks?position=chr13:47469690-47470190&hgsid=247045277&snp135Common=pack&hgFind.matches=rs6313,) | CT | 0.75 | -0.01 | 0.8526 | -0.04 | 0.5235 | 0.03 | 0.7170 | -0.01 | 0.8846 | -0.04 | 0.4150 | -0.01 | 0.9119 | 0.01 | 0.9033 | -0.04 | 0.5302 | 0.02 | 0.1874 | 0.03 | 0.7982 |
|  | rs6311 | [chr13:47471228-47471728](http://genome.ucsc.edu/cgi-bin/hgTracks?position=chr13:47471228-47471728&hgsid=247045277&snp135Common=pack&hgFind.matches=rs6311,) | CT |  | -0.01 | 0.8177 | -0.04 | 0.5488 | 0.02 | 0.8220 | 0.01 | 0.9145 | -0.01 | 0.8628 | 0.02 | 0.7876 |  |  | -0.00 | 0.9340 | 0.02 | 0.1165 | -0.01 | 0.9514 |
| ***HTR2C*** |  | chrX:113818551-114144624 |  |  |  |  |  |  |  |  |  |  |  |  |  |  |  |  |  |  |  |  |  |  |
|  | rs3813929 | [chrX:113818270-113818770](http://genome.ucsc.edu/cgi-bin/hgTracks?position=chrX:113818270-113818770&hgsid=247045277&snp135Common=pack&hgFind.matches=rs3813929,) | CT | 0.32 | -0.06 | 0.4057 | -0.09 | 0.2472 | -0.02 | 0.8184 | 0.06 | 0.2739 | 0.08 | 0.1874 | -0.02 | 0.8241 | 0.08 | 0.2418 | -0.05 | 0.4666 | -0.01 | 0.7224 | 0.14 | 0.2192 |
|  | rs518147 | [chrX:113818332-113818832](http://genome.ucsc.edu/cgi-bin/hgTracks?position=chrX:113818332-113818832&hgsid=247045277&snp135Common=pack&hgFind.matches=rs518147,) | CG | 0.42 | 0.01 | 0.8889 | 0.05 | 0.3869 | -0.05 | 0.4208 | 0.07 | 0.0852 | 0.07 | 0.1047 | 0.02 | 0.7302 | 0.09 | 0.0786 | -0.03 | 0.5722 | -0.00 | 0.7845 | 0.12 | 0.1473 |
|  | rs6318 | [chrX:113965485-113965985](http://genome.ucsc.edu/cgi-bin/hgTracks?position=chrX:113965485-113965985&hgsid=247045277&snp135Common=pack&hgFind.matches=rs6318,) | CysSer(=GC) |  | 0.03 | 0.6182 | 0.10 | 0.1135 | -0.06 | 0.3484 | 0.05 | 0.3115 | 0.04 | 0.3519 | 0.04 | 0.4749 | 0.05 | 0.3905 | -0.00 | 0.9433 | -0.00 | 0.9808 | 0.05 | 0.5607 |
| ***COMT*** |  | chr22:19929263-19957496 |  |  |  |  |  |  |  |  |  |  |  |  |  |  |  |  |  |  |  |  |  |  |
|  | rs4680 | [chr22:19951021-19951521](http://genome.ucsc.edu/cgi-bin/hgTracks?position=chr22:19951021-19951521&hgsid=247045277&snp135Common=pack&hgFind.matches=rs4680,) | GA | - | 0.03 | 0.5685 | 0.00 | 0.9705 | 0.07 | 0.3059 | 0.09 | 0.0580 | 0.08 | 0.1234 | 0.14 | 0.0303 | 0.09 | 0.1304 | 0.13 | 0.0289 | -0.00 | 0.9573 | 0.03 | 0.7503 |
| ***MnSOD*** |  | chr6:160100151-160114353 |  |  |  |  |  |  |  |  |  |  |  |  |  |  |  |  |  |  |  |  |  |  |
|  | rs4880 | [chr6:160113622-160114122](http://genome.ucsc.edu/cgi-bin/hgTracks?position=chr6:160113622-160114122&hgsid=247045277&snp135Common=pack&hgFind.matches=rs4880,) | TC | - | -0.01 | 0.8334 | -0.06 | 0.3705 | 0.04 | 0.5418 | -0.05 | 0.2605 | -0.00 | 0.9284 | -0.11 | 0.0830 | -0.06 | 0.2913 | -0.02 | 0.7653 | -0.03 | 0.0399 | -0.16 | 0.1008 |
| ***CYP1A2*** |  | chr15:75041184-75048941 |  |  |  |  |  |  |  |  |  |  |  |  |  |  |  |  |  |  |  |  |  |  |
|  | rs2069514 | [chr15:75037970-75038470](http://genome.ucsc.edu/cgi-bin/hgTracks?position=chr15:75037970-75038470&hgsid=247045277&snp135Common=pack&hgFind.matches=rs2069514,) | GA | 0.00 | -0.05 | 0.8504 | 0.12 | 0.7115 | -0.28 | 0.3956 | 0.06 | 0.8057 | 0.14 | 0.5826 | 0.16 | 0.6095 | -0.01 | 0.9601 | 0.13 | 0.6587 | 0.03 | 0.6051 | -0.11 | 0.8089 |
|  | rs762551 | [chr15:75041667-75042167](http://genome.ucsc.edu/cgi-bin/hgTracks?position=chr15:75041667-75042167&hgsid=247045277&snp135Common=pack&hgFind.matches=rs762551,) | AC |  | -0.08 | 0.2251 | -0.09 | 0.2221 | -0.07 | 0.3561 | -0.04 | 0.4522 | -0.06 | 0.3394 | -0.01 | 0.9252 | -0.04 | 0.5337 | 0.06 | 0.3912 | -0.03 | 0.0586 | -0.08 | 0.4910 |
| ***RGS2*** |  | chr1:192778169-192781406 |  |  |  |  |  |  |  |  |  |  |  |  |  |  |  |  |  |  |  |  |  |  |
|  | rs1933695 | [chr1:192764570-192765070](http://genome.ucsc.edu/cgi-bin/hgTracks?position=chr1:192764570-192765070&hgsid=247045277&snp135Common=pack&hgFind.matches=rs1933695,) | GA | 0.24 | 0.07 | 0.3177 | 0.05 | 0.5356 | 0.11 | 0.2027 | 0.00 | 0.9525 | 0.05 | 0.4633 | 0.03 | 0.6723 | -0.03 | 0.7222 | 0.08 | 0.2841 | -0.00 | 0.8287 | -0.06 | 0.6221 |
|  | rs2179652 | [chr1:192769576-192770076](http://genome.ucsc.edu/cgi-bin/hgTracks?position=chr1:192769576-192770076&hgsid=247045277&snp135Common=pack&hgFind.matches=rs2179652,) | TC | 0.27 | -0.07 | 0.2519 | -0.08 | 0.2248 | -0.06 | 0.4446 | -0.00 | 0.9888 | 0.01 | 0.8849 | 0.02 | 0.7898 | -0.01 | 0.8271 | -0.09 | 0.1269 | 0.00 | 0.8772 | 0.04 | 0.6706 |
|  | rs2746073 | [chr1:192778977-192779477](http://genome.ucsc.edu/cgi-bin/hgTracks?position=chr1:192778977-192779477&hgsid=247045277&snp135Common=pack&hgFind.matches=rs2746073,) | TA | 1.00 | 0.00 | 0.9737 | 0.07 | 0.3861 | -0.08 | 0.2992 | 0.01 | 0.9238 | -0.03 | 0.6777 | -0.03 | 0.6939 | 0.03 | 0.6512 | 0.05 | 0.4383 | -0.01 | 0.5270 | -0.07 | 0.5565 |
|  | rs4606 | [chr1:192780922-192781422](http://genome.ucsc.edu/cgi-bin/hgTracks?position=chr1:192780922-192781422&hgsid=247045277&snp135Common=pack&hgFind.matches=rs4606,) | CG | 1.00 |  |  |  |  |  |  |  |  |  |  |  |  |  |  |  |  |  |  |  |  |
|  | rs1819741 | [chr1:192784588-192785088](http://genome.ucsc.edu/cgi-bin/hgTracks?position=chr1:192784588-192785088&hgsid=247045277&snp135Common=pack&hgFind.matches=rs1819741,) | TC | 0.00 |  |  |  |  |  |  |  |  |  |  |  |  |  |  |  |  |  |  |  |  |
|  | rs1152746 | [chr1:192796655-192797155](http://genome.ucsc.edu/cgi-bin/hgTracks?position=chr1:192796655-192797155&hgsid=247045277&snp135Common=pack&hgFind.matches=rs1152746,) | AG |  | 0.02 | 0.7913 | -0.03 | 0.6875 | 0.08 | 0.3095 | -0.01 | 0.9194 | 0.03 | 0.6062 | -0.03 | 0.6798 | -0.01 | 0.8803 | 0.04 | 0.5195 | 0.03 | 0.0456 | 0.02 | 0.8525 |

Sources: UCSC (GRCh37/hg19), NCBI, SNPedia, Genecards, CHIP Bioinformatics Tools

^a^ Linkage disequilibrium (LD) measured in r^2^ for all SNPs located next to each other

^b^ TD=tardive dyskinesia, OF=orofacial dyskinesia, LT=limb truncal dyskinesia, PK=parkinsonism, RT=rest tremor, RG=rigidity, BK=bradykinesia, AK=akathisia, TDt=tardive dystonia and PF=principal-factor
